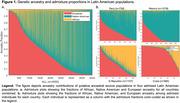# Social determinants of health and not global genetic ancestry predicts cognitive performance and dementia risk in Latinos

**DOI:** 10.1002/alz.086626

**Published:** 2025-01-03

**Authors:** Jorge J. Llibre‐Guerra, Miao Jiang, Isaac Acosta‐Castillo, Ana Luisa Sosa Ortiz, Daisy M Acosta, Ivonne Z. Jimenez Velazquez, Mariella Guerra, Aquiles Salas, Ana M Rodriguez‐Salgado, Juan C Llibre Guerra, Nedelys Díaz Sánchez, Matthew Prina, Alan E. Renton, Jennifer S. Yokoyama, Juan J. Llibre‐Rodriguez, 1066 Dementia Research Group

**Affiliations:** ^1^ Washington University in St. Louis, School of Medicine, St. Louis, MO USA; ^2^ Institute of Public Health, Faculty of Biomedical Sciences, Università della Svizzera italiana, Lugano Switzerland; ^3^ Dementias Laboratory, National Institute of Neurology and Neurosurgery, Mexico City, DF Mexico; ^4^ Instituto Nacional de Neurología y Neurocirugía, Mexico Mexico; ^5^ National Institute of Neurology and Neurosurgery, Mexico City Mexico; ^6^ Universidad Nacional Pedro Henriquez Ureña, Santo Domingo, Distrito Nacional Dominican Republic; ^7^ University of Puerto Rico, School of Medicine, San Juan, Puerto Rico, PR USA; ^8^ Universidad Peruana Cayetano Heredia, Lima, Lima Peru; ^9^ Medicine Department, Caracas University Hospital, Faculty of Medicine, Universidad Central de Venezuela, Caracas, Caracas Venezuela (Bolivarian Republic of); ^10^ Global Brain Health Institute, San Francisco, CA USA; ^11^ Department of Neurology, Salamanca Hospital, Salamanca, Salamanca Spain; ^12^ Dementia Research Unit, Facultad de Medicina Finlay‐Albarran, Medical University of Havana, Habana Cuba; ^13^ Newcastle University, Newcastle United Kingdom; ^14^ Ronald M. Loeb Center for Alzheimer’s Disease, Friedman Brain Institute, Icahn School of Medicine at Mount Sinai, New York, NY USA; ^15^ Memory and Aging Center, UCSF Weill Institute for Neurosciences, University of California, San Francisco, San Francisco, CA USA; ^16^ Dementia Research Unit/Medical University of Havana, Havana, Havana Cuba

## Abstract

**Background:**

Alzheimer’s disease and related dementias (ADRD) disproportionately affect Latinos compared to non‐Latino whites. Leveraging the non‐monolithic structure of Latin America, which represents a large variability in social determinants of health (SDoH) and high levels of genetic admixture, we aimed to determine contributors to ADRD disparities within Latinos, focusing on genetic ancestry and SDoH.

**Method:**

Community‐dwelling participants aged 65 and older (n = 4000) from Cuba, Dominican Republic, Mexico, and Peru completed the 10/66 protocol assessments, including sociodemographic and risk factors questionnaire, neurological exam, cognitive assessment, and blood draw. Dementia was diagnosed using the cross‐culturally validated 10/66 algorithm. Individual admixture proportions were determined using sixty ancestry‐informative markers. To evaluate the effect of global ancestry on dementia and cognitive performance, we used multivariate linear regression models adjusted for SDoH (e.g., gender, education level, socioeconomic status (SES), rural area, and vascular risk factors).

**Result:**

We observed extensive three‐way (African/European/Native American) genetic ancestry variation between countries (Figure 1). Individuals with higher proportions of Native American (>70%) and African American (>70%) ancestry were more likely to exhibit factors contributing to worse SDoH, such as lower educational levels (p < 0.001), lower SES (p < 0.001), and higher frequency of vascular risk factors (p < 0.001). As a result, in unadjusted analysis, individuals with predominant African ancestry exhibited a higher dementia frequency (p = 0.03) and both Native American and African ancestry predominant groups showed lower cognitive performance relative to those with higher European ancestry (p<0.001). However, after adjusting for measures of SDoH, there was no association between ancestry proportion and dementia probability, and ancestry proportions no longer significantly accounted for the variance in cognitive performance (African predominant p = 0.31 [‐0.19, 0.59] and Native predominant p = 0.74 [‐0.24, 0.33]).

**Conclusion:**

We report a comprehensive characterization of the role of global genetic ancestry in cognitive performance and dementia in Latin America while controlling for SDoH. In our study, adjustment of SDoH attenuated associations between genetic ancestry, dementia probability, and cognitive performance. These findings highlight that social and environmental factors likely play more critical roles than genetic ancestry in determining racial/ethnic disparities in cognitive performance and subsequent dementia risk.